# LocSigDB: a database of protein localization signals

**DOI:** 10.1093/database/bav003

**Published:** 2015-02-27

**Authors:** Simarjeet Negi, Sanjit Pandey, Satish M. Srinivasan, Akram Mohammed, Chittibabu Guda

**Affiliations:** ^1^Department of Genetics, Cell Biology and Anatomy, ^2^Bioinformatics and Systems Biology Core, ^3^Department of Biochemistry and Molecular Biology, ^4^Fred and Pamela Buffet Cancer Center and ^5^Eppley Institute for Research in Cancer and Allied Diseases, University of Nebraska Medical Center, Omaha, NE 68198, USA

## Abstract

LocSigDB (http://genome.unmc.edu/LocSigDB/) is a manually curated database of experimental protein localization signals for eight distinct subcellular locations; primarily in a eukaryotic cell with brief coverage of bacterial proteins. Proteins must be localized at their appropriate subcellular compartment to perform their desired function. Mislocalization of proteins to unintended locations is a causative factor for many human diseases; therefore, collection of known sorting signals will help support many important areas of biomedical research. By performing an extensive literature study, we compiled a collection of 533 experimentally determined localization signals, along with the proteins that harbor such signals. Each signal in the LocSigDB is annotated with its localization, source, PubMed references and is linked to the proteins in UniProt database along with the organism information that contain the same amino acid pattern as the given signal. From LocSigDB webserver, users can download the whole database or browse/search for data using an intuitive query interface. To date, LocSigDB is the most comprehensive compendium of protein localization signals for eight distinct subcellular locations.

**Database URL**: http://genome.unmc.edu/LocSigDB/

## Introduction

Proteins are synthesized in the cytoplasm and on ribosomes bound to the endoplasmic reticulum (ER), with a few proteins synthesized in mitochondria or chloroplasts (as in plant cells). However, the nucleus-encoded proteins are targeted to different subcellular locations to carry out defined functions. Aberrant mislocalization of proteins to unintended locations can interfere with numerous cellular processes, often leading to many diseases (1). Proteins are directed to their appropriate destinations by a process called protein targeting or protein sorting, which is primarily dependent upon the information contained in the targeted protein itself; known as ‘intrinsic signals’ or ‘address tags’ (2, 3). These signals are stretches of amino acid residues within a protein; they are present either at the N-terminus; as is the case in Golgi, the secretory pathway or mitochondrial proteins (4–6), or at the C-terminus; as in the case of peroxisomes and ER (7, 8). Signals at the N-terminus are usually referred to as signal peptides and they help direct proteins to cellular as well as extracellular locations. Generic structure of a signal peptide consists of a positively charged N-terminus followed by a long stretch of hydrophobic region at the core. The C-terminus amino acid composition varies depending upon the organelle the protein enters or is inserted into (in case of cellular membranes) or if the protein is secreted out of the cell. However, there are exceptions to these general rules: Golgi retention signals can be found at the C-terminus (9) and mitochondrial signal peptides can also be found at the internal positions (10) or at the C-terminus (11) of some proteins. Conversely, the nucleus has its own class of targeting signals (12, 13), which can be located anywhere on the peptide chain and usually contain basic, positively charged amino acids. The precise functioning of cells and tissues relies on the fidelity of protein targeting. Consequently, diseases like cancer and psoriasis and inflammatory conditions such as sepsis, rheumatoid arthritis and tissue rejection, can result from the malfunction of signalling pathways (14, 15). Furthermore, errors in these address tags can result in heritable diseases (16). As a result, the biomedical community will greatly benefit form a catalogue of experimentally identified protein subcellular localization signals as a way to manage and manipulate diseases by exploitation of these sorting signals (17–19).

Many computational methods have been developed for the prediction of protein subcellular localization (20); however, there was little or no emphasis on predicting the sorting signals. Some methods that predict the sorting signals limit their predictions to N-terminal signal peptides and their cleavage sites (21, 22). Likewise, few methods predict the internal nuclear localization signals (23, 24) and also C-terminal peroxisomal targeting signals (25), but again these methods are limited to single organelles. Also potential sorting signals are identified by LOCATE database based on PROSITE patterns and signal peptide prediction methods; however; it does not document any experimental confirmation of these signals (26). Similarly, databases that are dedicated to protein subcellular localization (26, 27) only house information on catalogs of proteins in different organelles and provide no information on the sorting signals. Among the existing localization signal databases (28, 29), NLSdb (28) contains manually curated experimentally validated nuclear localization signals but, it accounts for only one-third of the currently available experimental nuclear localization signals that are catalogued in our LocSigDB (refer to Database Statistics Section). As for SPdb (29), it is a database of experimentally and computationally predicted localization signals, but limited to only signal peptides. Moreover, both the databases have not been updated in the recent years. Also ELM (30), a database of eukaryotic linear motifs is an excellent resource on functional sites in proteins including targeting motifs. However, the number of ELMs classified as targeting motifs is relatively few. Another rich source of sorting signals information is UniProt (31), which offers information on experimental as well as predicted (potential, probable or similarity based) sorting signals; however, the criterion for a signal to be identified as being experimental was not as rigorous as reported by LocSigDB, where evidence for every entry is backed by at least one PubMed article. But, beginning of September 2014, UniProt has also begun to adopt the evidence ontology combined with source information and for the experimental evidence codes; this source information is most often in the form of a PubMed ID. This has resulted in fewer sorting signals than before being classified as ‘experimental’. Other than the protein localization signal databases, a few interesting review articles have also been published (14, 32, 33) which catalog the experimentally validated localization signals, although the proportion of signals reported is much smaller than LocSigDB and typically the reviews are focussed either on a single organelle or on a protein of interest. Therefore, there is a need for developing an up-to-date and comprehensive database of experimentally known protein localization signals for all the major subcellular locations of a cell.

Over the past decade, we have developed a variety of tools for predicting protein subcellular localization (34–38). This has motivated us to develop the LocSigDB web server, which we believe will fill in the current gap. LocSigDB is a unique, extensive and substantially large database when compared to any existing localization signal databases. LocSigDB comprises sorting signal information for 533 distinct experimentally validated signals, along with the proteins that harbor them for eight distinct subcellular locations. We believe that LocSigDB will act as a value-adding resource for the biomedical research community to learn about the localization signals that have already been identified, as well as help form a rationale to deduce new potential signals.

## Aims of the database

LocSigDB is a database dedicated to protein subcellular localization signals and the proteins that harbor them, along with the research articles that have experimentally confirmed these signals. The main goal of this database is to collect and organize the information on localization signals and present it in a user-friendly and searchable format to facilitate easy retrieval of information. Our second goal is to ensure that the information in this database is up-to-date. In addition to monitoring the new literature, we also provide a signal submission form on our website for users to submit experimentally characterized sorting signals. We will review this information for authenticity before adding it to our database. We anticipate LocSigDB will serve as a comprehensive resource of protein targeting signals to the scientific community.

## Database content

### Collection of localization signals from literature

Over 1000 published articles related to protein-targeting signals were collected and reviewed to extract experimentally determined subcellular localization signals across eight major cellular compartments. Various keywords and their combinations were used in PubMed searches to retrieve appropriate literature; some examples are ‘Nuclear localization signal’, ‘NLS’, ‘Nuclear localization sequence’, ‘Mitochondrial targeting signal’, ‘Lysosome sorting signal’, and so forth. The returned literature was reviewed with a focus on the following criteria for selecting a signal as a valid sorting signal: (i) if a particular signal is able to target a non-resident protein to its own specific organelle (e.g. a signal is a valid nuclear localization signal if it can target a non-nuclear protein to nucleus), or (ii) by deleting or mutating some amino acids from the signal prevents import of the protein into its native subcellular location. Based on these criteria, only 518 articles were selected out of the 1000 articles that contained relevant information (some articles had information on more than one protein). Note that some researchers report a part of the protein sequence as a signal while others report rather a specific peptide sequence or a sequence pattern with a set of conserved residues or non-specific residues allowed in the patterns. Such signal patterns were translated into regular expressions in our database to facilitate pattern-based querying.

## Database fields

Each entry in the LocSigDB annotates a localization signal with five descriptors: (i) ***Protein(s):*** The protein(s) in which the experimental localization signal was reported in the literature. (ii) ***Localization:*** The exclusive subcellular location where the protein containing the targeting signal is found. (iii) ***Reference(s):*** Published primary literature on the experimental localization signals. This field has URLs that cross-link to PubMed citations. (iv) ***Coordinate(s):*** The start and end positions of the signals found is a given sequence. This parameter is displayed only during search by ‘Protein ID' or ‘Protein Sequence'. (v) ***UniProt Accession(s):*** UniProt accessions of all proteins that contain the same amino acid pattern as that of a given signal. Preferably SwissProt accessions for the protein are presented but in few cases the signals could not be mapped onto SwissProt annotated proteins; there the TrEMBL accessions have been reported. This helps achieve complete integration with the UniProt. (vi) ***Organisms:*** The organism(s) where each signal is found and this information is retrieved from the UniProt as it corresponds to the UniProt accessions and the respective organism representing the same. But the organism field is unique such that each organism is reported only once although more than one protein sequences (accessions) from the same organism may contain the signal of interest.

## Database statistics

**Figure 1.**
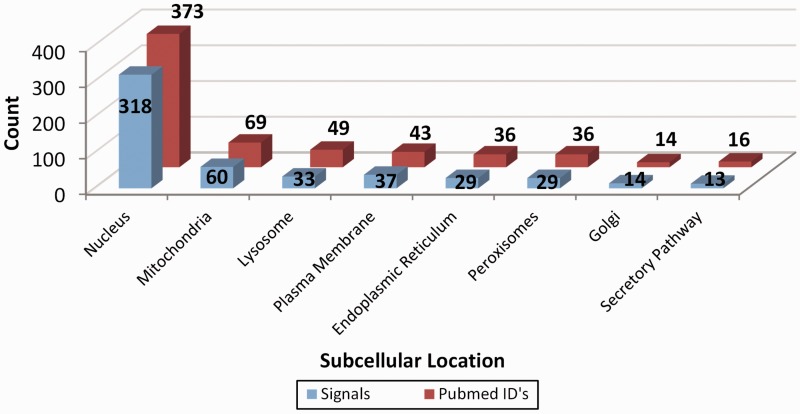
Representation of the database statistics showing the number of localization signals for distinct subcellular location along with the count of the research articles elucidating these signals. As clearly seen, most studies have been done on inferring the protein localization signals for nucleus followed by mitochondria and all the other six organelles with a wide margin.

**Figure 2.**
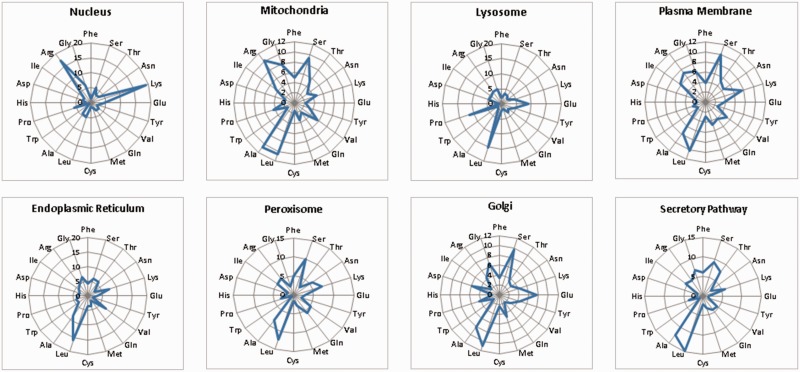
An overview of the frequency distribution of amino acids in the signal set for each of the eight subcellular organelles. As seen from the radar plots, there are clear differences in the frequency occurrence of amino acids for each distinct organelle. Nucleus is dominated by positively charged residues like lysines and arginines; whereas, mitochondrial signals have frequent occurrence of hydrophobic amino acids like: glycine, leucine and alanine as well as positively charged amino acids like arginine. Also, negatively charged amino acids like aspartic acid and glutamic acid are present only in the signals of organelles like Golgi and ER.

LocSigDB contains 533 experimentally validated localization signals. Figure 1 illustrates LocSigDB statistics with the number of localization signals and published articles for each distinct subcellular location. It can be observed from Figure 1, that the maximum number of studies have been done for inferring the localization signals for nucleus targeted proteins followed by mitochondria with a wide margin. Nuclear localization signals for 318 proteins accounted for a higher total number of signals than that of the remaining seven organelles combined. In comparison, the number of nuclear localization signals reported by NLSdb (28) accounts for only about one-third of those that are catalogued in LocSigDB. Organelle based radar plots were generated to look at the frequency distribution of each amino acid in the localization signals for each distinct subcellular location as shown in Figure 2. Here, the frequency of occurrence of an amino acid has been normalized by the number of experimentally verified localization signals as well as the average length of signals for each organelle to correlate the significance of certain amino acids in subcellular localization. In case of nuclear signals, it is evident that basic positively (39) charged amino acids, lysine (Lys) and arginine (Arg), significantly contribute in the nuclear localization of a protein. Similarly, mitochondrial signals have alternating hydrophobic and positively charged amino acids (40), which is evident from Figure 2; Mitochondrial signals are dominated by the frequent occurrence of hydrophobic amino acids leucine (Leu) and alanine (Ala), as well as positively charged amino acids like arginine (Arg) and small amino acids like glycine (Gly) and serine (Ser). Conversely, negatively charged amino acids like aspartic acid (Asp) and glutamic acid (Glu) are present in modest frequencies only in the localization signals of Golgi and ER. This observation is also consistent with the most common sequence patterns like KDEL and its related amino acid patterns (41). The most abundant amino acids: serine (Ser), leucine (Leu), alanine (Ala) and glycine (Gly), (42, 43) are prevalent in most of the localization signals except nuclear signals; while the least abundant amino acids: tryptophan (Trp), histidine (His) and cysteine (Cys) are present in lowest frequencies in the localization signals of all the eight subcellular locations.

## Database access and user interface

**Figure 3.**
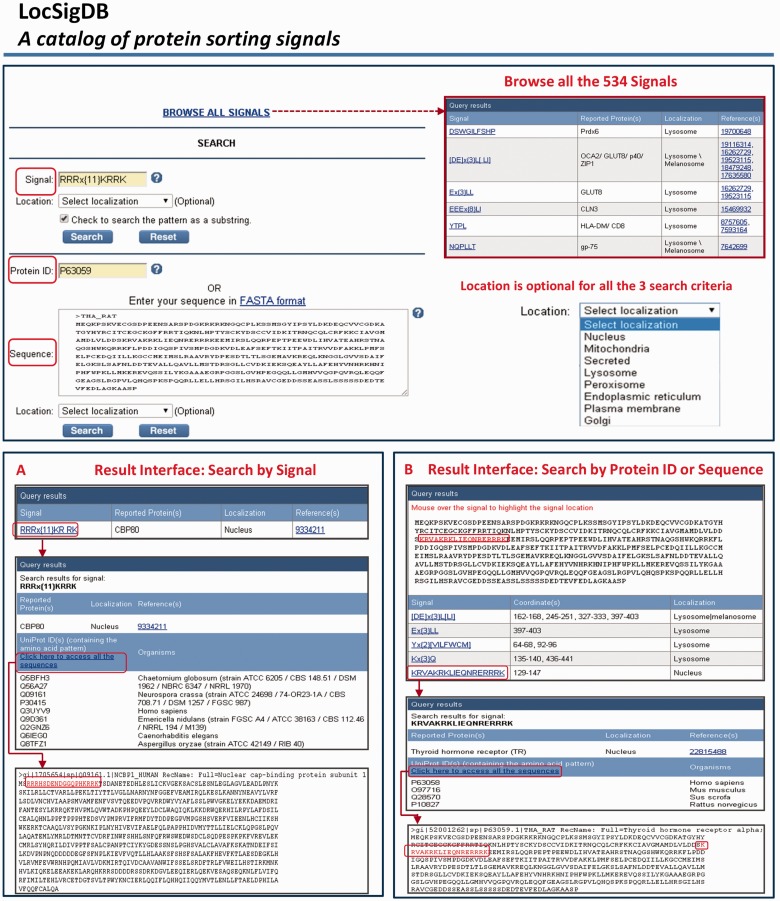
An overview of the query and the search results interface in LocSigDB. LocSigDB provides three search functions to retrieve the localization signal information. (**A**) Query using a signal displays links to all the available descriptors for the signal in question; (**B**) Query by a protein ID or protein sequence (FASTA format) will retrieve corresponding sequence from the public database. The sequence is displayed in the results interface and the user can mouse over the corresponding signals identified in the protein, while highlighting the signal coordinates in red on the protein sequence. Each signal is in turn linked to the signal attribute window that displays the annotations for that signal.

LocSigDB is freely accessible on the web at http://genome.unmc.edu/LocSigDB/. At this time, the database contains 533 experimentally identified protein localization signals, the proteins that harbour such signals and corresponding literature associated with each signal. LocSigDB provides three search functions to retrieve information pertaining to localization signals. Users can search the database by submitting (i) a defined localization signal or a motif pattern (using wild card characters), (ii) a protein identifier (NCBI’s RefSeq ID or UniProt’s protein accession or ID) or (iii) a protein sequence itself (in FASTA format). The web interface system is built using LAMP (Linux, Apache HTTP Server, MySQL and Perl) architecture. The data is stored in a MySQL database and is made available using a Perl/CGI backed user interface. When a user queries for a signal, the server searches for the signal in the LocSigDB and displays the results. The results page also contains links to further probe the information on signal motifs and PubMed citations. If the user wants to search using an NCBI protein identifier, the server automatically retrieves the sequence from the NCBI database using NCBI E-utilities and displays the results for the retrieved sequence. Similarly, a search can be made using UniProt/SwissProt IDs. In the third search option, a user can provide the full or a partial protein sequence in FASTA format. Certain sequence patterns (such as short repeats or common motifs) occur more frequently in protein sequences than others; thus, have a higher chance of randomly matching as substrings to a query pattern and generating false positives. Hence, we have made the substring search as ‘optional’ below the search window and the substring matches are displayed only if chosen by the user. Additionally, avoiding the use of wild character (*) or lax regex patterns in the queries will reduce the false positive matches. An overview of the query and result interface is shown in Figure 3, which demonstrates a step-by-step navigation through the database using relevant examples (given in the FAQs file) for the three query functions. The entire database can be downloaded as a tab-delimited, comma-delimited or as an excel file using the ‘Download’ link provided on the left panel. Users can browse the database contents and, highlight the special features, like signal highlights in the results page.

## Data submission

To encourage users to submit experimentally validated new localization signals/motifs, we have provided a ‘Submit signal’ link on the left panel. This link leads to a preformatted form for submission of signals on the LocSigDB webpage. We will review these signals and the literature for accuracy before including in our database. We intend to maintain and frequently update LocSigDB.

## Conclusion and future direction

We present LocSigDB, a comprehensive database and web interface to search and explore localization signals for eight distinct subcellular locations in eukaryotic and bacterial cells. LocSigDB has been designed to help better understand the signal-dependent transport of various proteins; and as a result it is intended to help the development of therapies based on the interception of cellular signals in diseased cells (15, 44). A number of research projects in our laboratory are tied to protein subcellular localization; therefore, we are motivated to keep this database up-to-date. The website will be updated twice a year, as new data become available and is thus intended to be a long-term resource for the research community in this area. Often, mutations in the localization signal can alter the subcellular location of a protein; consequently, we also plan to add disease causing mutated signals to the database in our next update. While the crux of LocSigDB is the experimentally verified signals, it can also be used as an important resource to develop new methodologies for predicting localization signals/motifs, which in turn can help advance the field. Based on the provided information, our long-term goal is to develop and maintain LocSigDB as the most comprehensive resource for protein localization signals.
